# Hepatocyte growth factor combined with adenosine deaminase as biomarker for diagnosis of tuberculous pleural effusion

**DOI:** 10.3389/fmicb.2023.1181912

**Published:** 2023-07-06

**Authors:** Sheng-Cai Zheng, Zhong-Yin Huang, Kan Zhai, Huan-Zhong Shi, Ming-Ming Shao

**Affiliations:** ^1^Department of Respiratory and Critical Care Medicine, Beijing Institute of Respiratory Medicine and Beijing Chao-Yang Hospital, Capital Medical University, Beijing, China; ^2^Clinical Center for Pleural Diseases, Capital Medical University, Beijing, China

**Keywords:** tuberculous pleural effusion, HGF, ADA, diagnostic efficacy, biomarkers

## Abstract

**Background:**

The simple, rapid, and accurate diagnosis of tuberculous pleural effusion (TPE) remains difficult. This study aimed to determine the accuracy of hepatocyte growth factor (HGF) in the diagnosis of TPE.

**Methods:**

We quantified the expression of HGF, adenosine deaminase (ADA), and interferon gamma (IFN-γ) in pleural effusion (PE) in 97 TPE subjects and 116 non-TPE subjects using an enzyme-linked immunosorbent assay (ELISA) or a fully automatic biochemical analyzer. The diagnostic performance of these three biomarkers was evaluated using a receiver operating characteristic (ROC) curve of subjects by age and gender.

**Results:**

We discovered that the TPE group had much higher levels of HGF than the non-TPE group, regardless of age or gender, and that there was no statistically significant difference between the two groups’ levels of HGF expression in peripheral plasma. In female TPE patients aged ≤65 years, the AUCs of TPE and non-TPE diagnosed by HGF, ADA or IFN-γ were 0.988, 0.964, and 0.827, respectively. HGF plus ADA had the highest diagnostic efficacy in female TPE patients aged ≤65 years. With HGF plus ADA having a cut-off value of 0.219 for distinguishing TPE from non-TPE, the area under the curve (AUC), sensitivity (SEN), specificity (SPE), positive predictive value (PPV), and negative predictive value (NPV) were, respectively, 0.998 (95% confidence interval [CI], 0.993–1.000), 100 (95% CI, 89.997–100.000), 96.667 (95% CI, 82.783–99.916), 97.222 (95% CI, 83.594–99.586), and 100.

**Conclusion:**

This study confirmed that HGF plus ADA has high diagnostic efficacy in younger female TPE patients and has the potential to be an excellent biomarker.

## Introduction

Tuberculosis (TB) is by far the leading cause of death from infectious disease worldwide, disproportionately affecting immunosuppressed patients and socioeconomically vulnerable populations (Chakaya et al., 2022). Tuberculous pleural effusion (TPE) is not uncommon clinically, but its diagnosis is difficult (Baumann et al., 2007; Pang et al., 2019; Kang et al., 2020). In the diagnosis of TPE, the available gold standards are as follows: (1) microscopic examination of Ziehl–Neelsen staining smear; (2) *Mycobacterium tuberculosis* (*Mtb*) culture detection in pleural effusion (PE); and (3) pleural biopsy (PB), followed by pathological microscopy. However, the detection rates of the first two methods are often low, while the last is an invasive examination with certain surgical risks and complications that has low patient acceptance. Biomarkers are a fast, simple diagnostic method (Porcel, 2016; Zhang et al., 2020); therefore, it is important to find reliable biomarkers of TPE such as adenosine deaminase (ADA). Originally, hepatocyte growth factor (HGF) was identified as a liver-regenerative circulatory factor and named for its increased protein expression levels after liver injury or hepatectomy (Nakamura et al., 1989; Sonnenberg et al., 1993; Zhao et al., 2022). Many studies have demonstrated that HGF has a variety of activities, including mitosis, morphogenesis, and motor effects in different tissues (Stoker et al., 1987; Montesano et al., 1991; Ebens et al., 1996). HGF is a cytokine that plays a pleiotropic role in inflammatory response and immune regulation (Li et al., 2022). Increasing evidence from *in vivo* animal experiments also supports the protective role of HGF in various immune inflammatory diseases, including classic autoimmune diseases such as rheumatoid arthritis (RA) and autoimmune neuroinflammation, and typical inflammatory diseases such as inflammatory bowel disease (IBD) and asthma. Taken together, this evidence suggests that HGF might play an important anti-inflammatory role (Wang M. et al., 2018).

Studies have also reported that HGF levels in PBMCs are significantly downregulated in TB patients compared with LTBI patients or healthy subjects (He et al., 2020). In addition, the expression of HGF in the serum of TB patients has been found to be higher than that in healthy subjects but lower than that in bacterial-pneumonia patients (Huang et al., 1999). One study found that HGF expression in the cerebrospinal fluid (CSF) of Alzheimer disease (AD) patients was higher than in that of patients with other neurological diseases; the AUC for the diagnosis of AD reached 0.802 (Gaetani et al., 2021). Other studies have shown that the level of HGF in the plasma of cirrhosis patients was higher than that in patients with liver fibrosis, with an AUC of 0.71 (Andersen et al., 2011). However, the expression of HGF in TPE and its diagnostic value have not been reported to date. In addition, research suggests that age and gender might play important roles in immune regulation (Markle and Fish, 2014; Giefing-Kröll et al., 2015; Jiang et al., 2020). Therefore, in this study, we analyzed subgroups using age and gender as criteria to determine the diagnostic efficacy of HGF in distinguishing TB patients with TPE from those non-TPE patients.

## Materials and methods

### Study populations and sample collection

The patients included in this study were admitted to the Department of Respiratory and Critical Care Medicine, Beijing Chaoyang Hospital, Capital Medical University (CMU; Beijing, China) from June 2019 to June 2022. The exclusion criteria were as follows: (1) any invasive pleural surgery or thoracic trauma within 3 months prior to hospitalization; (2) any anti-tubercular chemotherapy, antitumor therapy, glucocorticoids, or other nonsteroidal anti-inflammatory treatment; (3) diabetes; and (4) COVID-19, HIV or latent TB infection. Given that activation of HGF is related to glucose abnormalities or the pathogenesis of diabetes and can affect HGF expression in PEs, we excluded patients with abnormal glucose metabolism or diabetes (Oliveira et al., 2018). Patients with confirmed TPE were included in this study; non-TPE cases included patients with malignant PE (MPE) and parapneumonic PE (PPE).

Clinically, TPE is confirmed if the PE or PB specimen is positive for *Mtb* culture, Ziehl–Neelsen staining is positive, or granuloma is found in pleural biopsy specimens. MPE is confirmed when malignant cells are found in PE and/or PB specimens. PPE is defined as being associated with pulmonary infections such as bacterial pneumonia, lung abscess, and bronchiectasis and as featuring the presence of PE.

In this study, we collected PEs and peripheral blood samples and centrifuged them at 400 g for 10 min. The supernatant was separated and stored at −80°C for subsequent detection of HGF, ADA, and interferon gamma (IFN-γ).

This study was approved by the Ethics Committee of Beijing Chao-Yang Hospital of CMU. All participants were fully informed about this study and signed informed-consent forms.

### Determination of HGF, ADA, and IFN-γ protein concentrations

We measured the levels of HGF and IFN-γ in PE using enzyme-linked immunosorbent assay (ELISA) kits (Thermo Fisher Scientific, Waltham, MA, USA) as per manufacturer’s instructions. The expression of ADA in PE was detected using a fully automatic biochemical analyzer (Toshiba Corp., Tokyo, Japan) as per manufacturer’s instructions. All samples were assessed in duplicate.

### Statistical analysis

Continuous statistics in this study are expressed as medians (25th–75th percentiles). Categorical data are described by frequency. If data did not conform to a normal distribution, we used the Mann–Whitney *U* test to compare differences in continuous statistics between groups and the *χ*^2^ test to compare categorical data. Receiver operating characteristic (ROC) analysis was used to determine the ability of HGF, ADA, and IFN-γ to distinguish TPE from non-TPE, expressed as the area under the curve (AUC) (Hanley and McNeil, 1982; Zweig and Campbell, 1993). We used GraphPad Prism version 8.0 (GraphPad Software, Inc., San Diego, CA, USA) and MedCalc 20.0.4 (MedCalc Software Ltd., Ostend, Belgium) for statistical analysis. *p* < 0.05 was considered statistically significant.

## Results

### Clinical and demographic characteristics of patients with pleural effusion

Some data on diagnostic types, complications, and demographics for TPE and non-TPE patient groups are shown in Table 1. Based on our preliminary results, we used 65 years as the cut-off value for the age subgroups: the younger group was ≤65 years of age, and the elderly group was >65 years of age. In this study, patients with TPE were younger overall than those without TPE (*p* < 0.0001).

**Table 1:** Baseline characteristics according to study population.

Variable	Total population	TPE (n = 97)	Non-TPE (n = 116)	*p*-value
Gender, male/female, n	131/82	58/39	73/43	0.639
Age, years	50 (35, 65)	35 (31, 45)	63 (52, 67)	<0.0001
Diagnosis				
tuberculosis		97	0	-
lung cancer		0	95	-
mesothelioma		0	1	-
pneumonia		0	20	-
Complication				
Chronic obstructive pulmonary disease		0	1	-
hypertension		0	7	-
hyperlipidemia		0	1	-

Data are presented as median (25th–75th centile). Differences of continuous data between groups were compared using Mann–Whitney U-test, and χ2-test was used for comparisons of categorical data. TPE, tuberculous pleural effusion; non-TPE, non-tuberculous pleural effusion.

### Expression of HGF, ADA, and IFN-γ in pleural effusion

**Figure 1:**
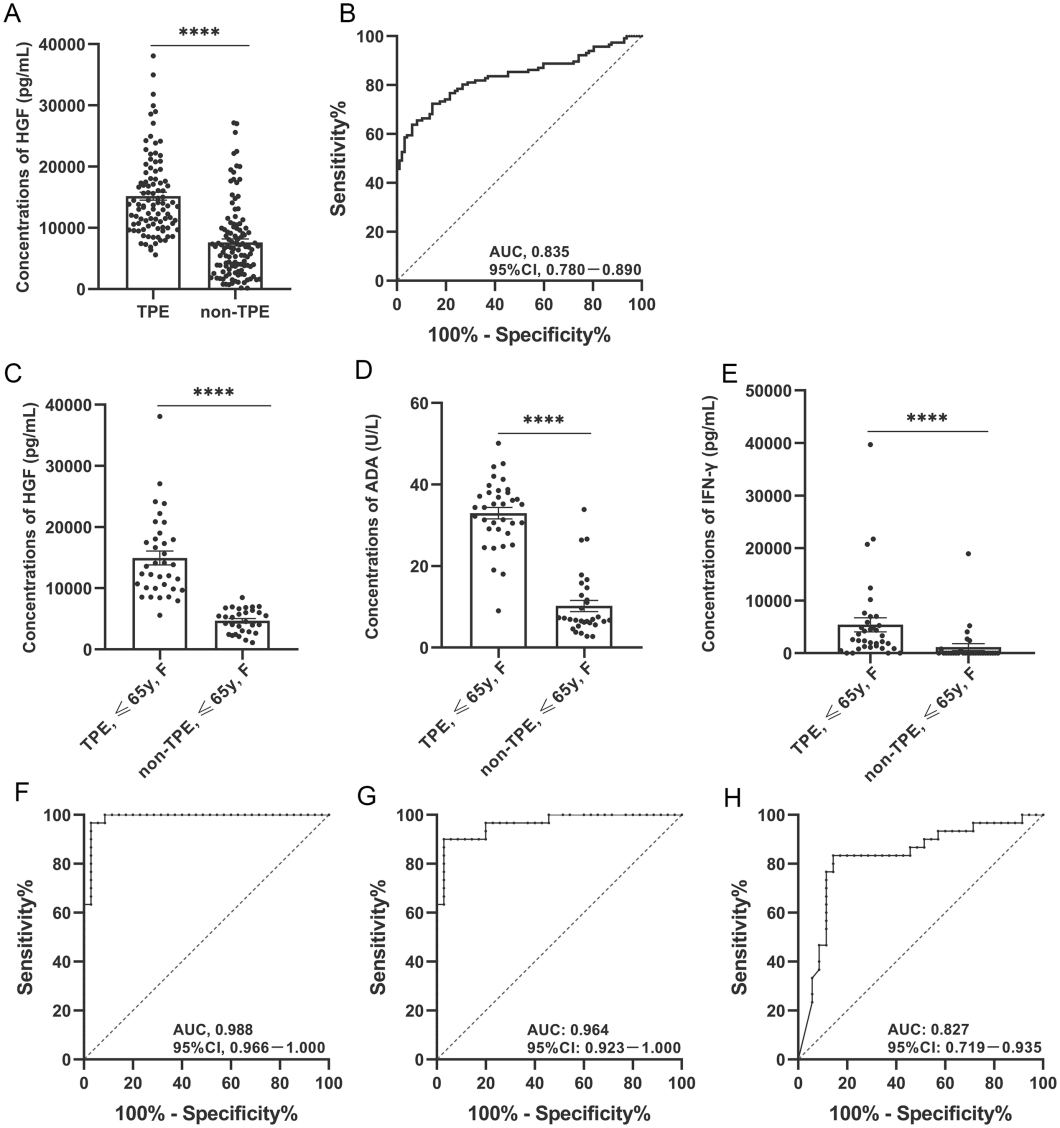
Diagnostic accuracy of HGF, ADA, and IFN-γ in PE for TPE overall or by gender. Expression of HGF in PE from TPE and non-TPE patients **(A)**; expression of HGF, ADA, and IFN-γ in PE from female TPE and non-TPE patients aged ≤65 years, respectively **(C–E)**. ROC curves show the diagnostic value of HGF in TPE and non-TPE patients **(B)**. ROC curves show the diagnostic value of HGF, ADA, and IFN-γ in female TPE and non-TPE patients aged ≤65 years **(F–H)**. *p < 0.05, **p < 0.01, ***p < 0.001, ****p < 0.0001. TPE, tuberculous pleural effusion; non-TPE, non-tuberculous pleural effusion. HGF, hepatocyte growth factor; ADA, adenosine deaminase; IFN-γ, interferon gamma. F, female.

Regardless of age or gender, the expression of HGF in the PE of the TPE group was higher than that in the non-TPE group, to a statistically significant degree (*p* < 0.0001; Table 2; Figure 1A). In addition, we found no statistically significant difference in HGF expression in peripheral plasma between the two groups (*p* > 0.05; Supplementary Figure 1A). Other indicators such as ADA and IFN-γ were similar in their differences between age and gender subgroups in the TPE and non-TPE groups (Table 2; Supplementary Figure 1).

**Table 2:** Concentrations of HGF, ADA, and IFN-γ in PF according to age and/or gender.

Variable	TPE	non-TPE	*p*-value
HGF, pg./mL			
All ages	13715.00 (10433.00, 17998.00)	6511.30 (3082.50, 9948.80)	<0.0001
M	13905.00 (10938.00, 18018.00)	7214.00 (2686.00, 9935.00)	<0.0001
F	13476.00 (9991.00, 18049.00)	6012.00 (3787.00, 10856.00)	<0.0001
Age ≤ 65 y	13834.00 (10458.00, 18291.00)	4392.00 (2497.00, 6930.00)	<0.0001
M	14007.00 (10726.00, 19253.00)	4051.00 (1840.00, 7492.00)	<0.0001
F	13587.00 (9991.00, 18049.00)	4901.00 (2822.00, 6447.00)	<0.0001
Age > 65 y	13476.00 (10284.00, 16098.00)	10695.00 (8805.00, 17920.00)	0.2569
ADA, U/L			
All ages	36.10 (28.40, 41.45)	8.60 (6.33, 12.80)	<0.0001
M	38.40 (28.45, 44.45)	9.60 (6.450, 13.00)	<0.0001
F	34.40 (27.50, 38.00)	7.30 (5.90, 12.80)	<0.0001
Age ≤ 65 y	36.80 (30.58, 42.10)	9.00 (7.00, 14.00)	<0.0001
M	39.60 (34.00, 46.50)	10.60 (7.30, 14.80)	<0.0001
F	34.40 (29.00, 38.00)	6.90 (5.99, 13.28)	<0.0001
Age > 65 y	24.00 (16.00, 30.00)	8.00 (5.50, 12.50)	0.0002
IFN-γ, pg./mL			
All ages	2346.00 (819.30, 5066.00)	2.12 (0.00, 6.78)	<0.0001
M	2027.00 (725.60, 42,670)	1.40 (0.00, 6.125)	<0.0001
F	2371.00 (883.70, 5875.00)	3.45 (0.00, 11.95)	<0.0001
Age ≤ 65 y	2510.00 (1126.00, 5269.00)	2.12 (0.00, 6.54)	<0.0001
M	2445.00 (1138.00, 5268.00)	1.76 (0.00, 4.44)	<0.0001
F	2575.00 (1088.00, 5875.00)	3.45 (0.78, 152.80)	<0.0001
Age > 65 y	592.20 (0.00, 1222.00)	1.58 (0.00, 7.15)	0.106

Data are presented as median (25th–75th centile). Differences between groups were compared using Mann–Whitney U-test for HGF, ADA, and IFN-γ. TPE, tuberculous pleural effusion; non-TPE, non-tuberculous pleural effusion. HGF, hepatocyte growth factor; ADA, adenosine deaminase; IFN-γ, interferon gamma. M, male; F, female.

### Diagnostic value of HGF in pleural effusion

In the overall cohort, with a cut-off value of 9314.84 pg./mL, the AUC, sensitivity (SEN), specificity (SPE), positive likelihood ratio (PLR), negative likelihood ratio (NLR), positive predictive value (PPV), and negative predictive value (NPV) of HGF expression in PE to discriminate between TPE and non-TPE cases were 0.835 (95% confidence interval [CI], 0.780–0.890; *p* < 0.001), 85.567, 71.552%, 3.008, 0.202, 71.552, and 85.567, respectively (Table 3; Figure 1B). Meanwhile, the AUCs of ADA and IFN-γ were 0.932 and 0.833, respectively (Supplementary Figures 1B,D).

**Table 3:** Diagnostic performance of HGF in PF in differentiating between patients with TPE and those with non-TPE according to age or/and gender.

Variable	Cut-off value (pg/mL)	AUC (95% CI)	Sensitivity (%)	Specificity (%)	PLR	NLR	PPV (%)	NPV (%)
HGF, all ages	>9314.84	0.835	85.567	71.552	3.008	0.202	71.552	85.567
			(76.971–91.879)	(62.429–79.541)	(2.228–4.060)	(0.123–0.332)	(65.076–77.247)	(78.276–90.702)
M	>10695.41	0.847	77.586	82.192	4.357	0.273	77.586	82.192
			(64.728–87.491)	(71.475–90.163)	(2.611–7.269)	(0.167–0.445)	(67.477–85.240)	(73.862–88.288)
F	>7023.44	0.811	97.436	67.442	2.993	0.038	73.077	96.667
			(86.524–99.935)	(51.456–80.924)	(1.941–4.615)	(0.005–0.266)	(63.769–80.717)	(80.557–99.510)
HGF, age ≤ 65 y	>8224.00	0.967	91.860	91.781	11.176	0.089	92.941	90.541
			(83.946–96.665)	(82.964–96.924)	(5.179–24.118)	(0.043–0.181)	(85.918–96.600)	(82.426–95.130)
M	>9223.83	0.950	86.275	90.698	9.275	0.151	91.667	84.783
			(73.745–94.299)	(77.865–97.407)	(3.624–23.736)	(0.076–0.303)	(81.126–96.570)	(73.554–91.777)
F	>7023.44	0.988	97.143	93.333	14.571	0.031	94.444	96.552
			(85.083–99.928)	(77.926–99.182)	(3.815–55.657)	(0.004–0.212)	(81.654–98.483)	(80.187–99.486)

AUC, area under the curve; PLR, positive likelihood ratio; NLR, negative likelihood ratio; PPV, positive predictive value; NPV, negative predictive value. TPE, tuberculous pleural effusion; non-TPE, non-tuberculous pleural effusion. HGF, hepatocyte growth factor; ADA, adenosine deaminase; IFN-γ, interferon gamma. M, male; F, female.

We also measured the diagnostic accuracy of HGF in the PE of different subgroups. With a cut-off value of 7023.44 pg./mL, the AUC of HGF in the younger female group to differentiate between TPE and non-TPE cases was 0.988 (95% CI, 0.966–1.000; *p* < 0.001), while its Sen, SPC, PLR, NLR, PPV, and NPV were 97.143, 93.333%, 14.571, 0.031, 94.444, and 96.552, respectively (Table 4; Figure 1F). The male group, female group, younger group, and younger male group had AUCs of 0.847 (95% CI, 0.781–0.913; Table 3; Supplementary Figure 1F), 0.811 (95% CI, 0.711–0.911; Table 3; Supplementary Figure 1L), 0.967 (95% CI, 0.942–0.991; Table 3; Supplementary Figure 2D), and 0.950 (95% CI, 0.910–0.990; Table 3; Supplementary Figure 2J), respectively. The diagnostic-accuracy parameters of ADA and IFN-γ in different subgroups are shown in Tables 4, 5 and in Supplementary Figure 2.

**Table 4:** Diagnostic performance of ADA in PF in differentiating between patients with TPE and those with non-TPE according to age or/and gender.

Variable	Cut-off value (U/L)	AUC (95% CI)	Sensitivity (%)	Specificity (%)	PLR	NLR	PPV (%)	NPV (%)
ADA, all ages	>17.80	0.932	93.814	84.483	6.046	0.073	83.486	94.231
			(87.022–97.696)	(76.589–90.536)	(3.942–9.273)	(0.034–0.160)	(76.724–88.576)	(88.229–97.267)
M	>20.60	0.922	89.655	86.301	6.545	0.120	83.871	91.304
			(78.831–96.108)	(76.247–93.231)	(3.656–11.717)	(0.056–0.257)	(74.389–90.300)	(83.037–95.749)
F	>17.80	0.951	94.872	88.372	8.159	0.058	88.095	95.000
			(82.676–99.373)	(74.917–96.115)	(3.568–18.659)	(0.015–0.225)	(76.391–94.421)	(83.064–98.660)
ADA, age ≤ 65 y	>23.00	0.938	93.023	86.301	6.791	0.081	88.889	91.304
			(85.431–97.397)	(76.247–93.231)	(3.807–12.113)	(0.037–0.176)	(81.769–93.451)	(82.839–95.805)
M	>23.10	0.927	94.118	86.047	6.745	0.068	88.889	92.500
			(83.758–98.770)	(72.068–94.702)	(3.201–14.214)	(0.023–0.206)	(79.151–94.400)	(80.344–97.383)
F	>17.80	0.964	97.143	90.000	9.714	0.032	91.892	96.429
			(85.083–99.928)	(73.471–97.888)	(3.315–28.464)	(0.005–0.220)	(79.457–97.077)	(79.582–99.468)

AUC, area under the curve; PLR, positive likelihood ratio; NLR, negative likelihood ratio; PPV, positive predictive value; NPV, negative predictive value. TPE, tuberculous pleural effusion; non-TPE, non-tuberculous pleural effusion. HGF, hepatocyte growth factor; ADA, adenosine deaminase; IFN-γ, interferon gamma. M, male; F, female.

**Table 5:** Diagnostic performance of IFN-γ in PF in differentiating between patients with TPE and those with non-TPE according to age or/and gender.

Variable	Cut-off value (pg/mL)	AUC (95% CI)	Sensitivity (%)	Specificity (%)	PLR	NLR	PPV (%)	NPV (%)
IFN-γ, all ages	>159.48	0.833	87.629	85.345	5.979	0.145	83.333	89.189
			(79.387–93.441)	(77.576–91.225)	(3.830–9.335)	(0.085–0.248)	(76.205–88.644)	(82.853–93.371)
M	>159.48	0.823	86.207	87.671	6.992	0.157	84.746	88.889
			(74.619–93.852)	(77.882–94.205)	(3.760–13.003)	(0.082–0.301)	(74.922–91.175)	(80.695–93.869)
F	>34.12	0.844	89.744	81.395	4.824	0.126	81.395	89.744
			(75.779–97.134)	(66.599–91.609)	(2.558–9.095)	(0.049–0.322)	(69.884–89.187)	(77.377–95.724)
IFN-γ, age ≤ 65 y	>149.60	0.852	90.698	82.192	5.093	0.113	85.714	88.235
			(82.491–95.898)	(71.475–90.163)	(3.097–8.376)	(0.058–0.221)	(78.487–90.798)	(79.354–93.604)
M	>149.60	0.869	92.157	86.047	6.605	0.091	88.679	90.244
			(81.119–97.822)	(72.068–94.702)	(3.131–13.934)	(0.035–0.235)	(78.782–94.294)	(78.180–95.981)
F	>793.66	0.827	85.714	80.000	4.286	0.179	83.333	82.759
			(69.743–95.194)	(61.433–92.286)	(2.069–8.879)	(0.078–0.410)	(70.705–91.196)	(67.647–91.680)

TPE, tuberculous pleural effusion; non-TPE, non-tuberculous pleural effusion. HGF, hepatocyte growth factor; ADA, adenosine deaminase; IFN-γ, interferon gamma. M, male; F, female. AUC, area under the curve; PLR, positive likelihood ratio; NLR, negative likelihood ratio; PPV, positive predictive value; NPV, negative predictive value.

### Diagnostic value of HGF plus ADA, HGF plus IFN-γ and ADA plus IFN-γ in pleural effusion

**Figure 2:**
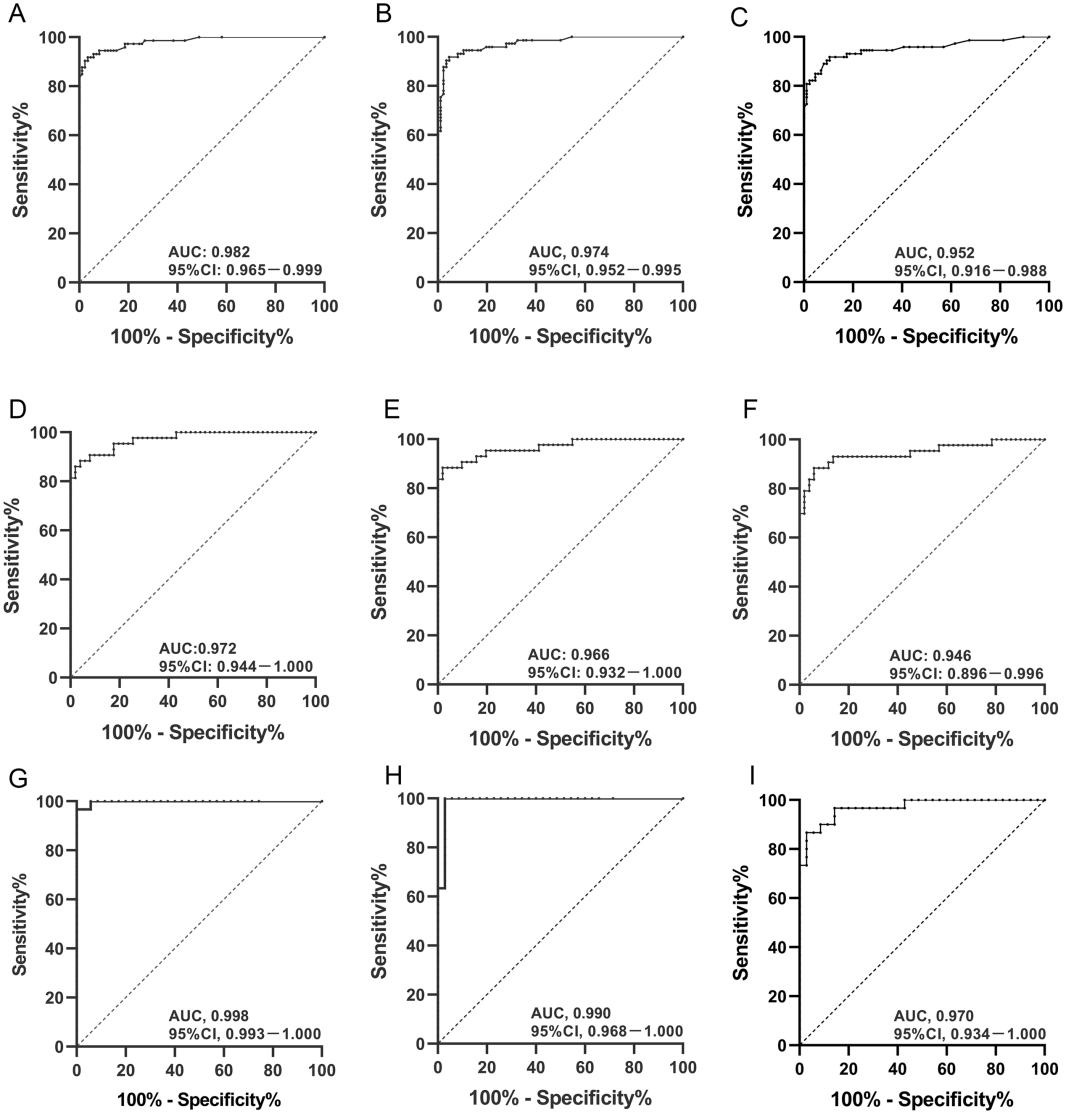
Diagnostic accuracy of HGF plus ADA, HGF plus IFN-γ, and ADA plus IFN-γ in PE for TPE by gender or age. AUC values of HGF plus ADA, HGF plus IFN-γ, and ADA plus IFN-γ in PE from TPE patients aged ≤65 years **(A–C)**; AUC values of HGF plus ADA, HGF plus IFN-γ, and ADA plus IFN-γ in PE from male TPE patients aged ≤65 years **(D–F)**; AUC values of HGF plus ADA, HGF plus IFN-γ, and ADA plus IFN-γ in PE from female TPE patients aged ≤65 years **(G–I)**. TPE, tuberculous pleural effusion. HGF, hepatocyte growth factor; ADA, adenosine deaminase; IFN-γ, interferon gamma.

In the age ≤ 65 years group, the AUC values of HGF combined with ADA, HGF combined with IFN-γ, and ADA combined with IFN-γ were 0.982, 0.974, and 0.952, respectively (Table 6; Figures 2A–C). In the males aged ≤65 years group, the AUCs of HGF plus ADA, HGF plus IFN-γ, and ADA plus IFN-γ were 0.972, 0.966, and 0.946, respectively (Table 7; Figures 2D–F). In the females aged ≤65 years group, the AUCs of HGF plus ADA, HGF plus IFN-γ, and ADA plus IFN-γ were 0.998, 0.990, and 0.970, respectively (Table 8; Figures 2G–I). In different subgroups, the AUCs of HGF plus ADA and of HGF plus IFN-γ were higher than those of each of HGF, ADA, and IFN-γ alone. In addition, the AUC of HGF plus ADA was higher than that of HGF plus IFN-γ or of ADA plus IFN-γ, especially in the females aged ≤65 years group, where we found that the AUC of HGF plus ADA peaked at 0.998 (Figure 2G). With a cut-off of 0.219, the SEN, SPE, PLR, NLR, PPV, and NPV of HGF plus ADA in the younger female group to differentiate between TPE and non-TPE cases were 100, 96.667%, 30, 0, 97.222, and 100%, respectively (Table 8).

**Table 6:** Diagnostic performance of HGF + ADA, HGF + IFN-γ, and ADA + IFN-γ in PF in differentiating between patients with TPE and those with non-TPE according to no more than 65 years.

	Variable	Cut-off value	AUC (95% CI)	Sensitivity (%)	Specificity (%)	PLR	NLR	PPV (%)	NPV (%)
Age ≤ 65 y	HGF + ADA	>0.319	0.982	96.512	91.781	11.742	0.038	93.258	95.714
			(0.965–0.999)	(90.14–99.28)	(82.96–96.92)	(5.450–25.300)	(0.012–0.116)	(86.523–96.754)	(88.00–98.55)
	HGF + IFN-γ	>0.397	0.974	95.349	91.781	11.601	0.051	93.182	94.366
			(0.952–0.995)	(88.517–98.718)	(82.964–96.924)	(5.382–25.005)	(0.019–0.132)	(86.377–96.717)	(86.518–97.764)
	ADA + IFN-γ	>0.652	0.952	89.535	91.781	10.893	0.114	92.771	88.158
			(0.916–0.988)	(81.061–95.102)	(82.964–96.924)	(5.044–23.527)	(0.061–0.212)	(85.595–96.518)	(79.987–93.273)

TPE, tuberculous pleural effusion; non-TPE, non-tuberculous pleural effusion. HGF, hepatocyte growth factor; ADA, adenosine deaminase; IFN-γ, interferon gamma. M, male; F, female. AUC, area under the curve; PLR, positive likelihood ratio; NLR, negative likelihood ratio; PPV, positive predictive value; NPV, negative predictive value.

**Table 7:** Diagnostic performance of HGF + ADA, HGF + IFN-γ, and ADA + IFN-γ in PF in differentiating between patients with TPE and those with non-TPE according to male with no more than 65 years.

	Variable	Cut-off value	AUC (95% CI)	Sensitivity (%)	Specificity (%)	PLR	NLR	PPV (%)	NPV (%)
Age ≤ 65 y, M	HGF + ADA	>0.404	0.972	96.078	88.372	8.263	0.044	90.741	95
			(0.944–1.000)	(86.541–99.522)	(74.917–96.115)	(3.618–18.871)	(0.011–0.173)	(81.100–95.723)	(82.944–98.671)
	HGF + IFN-γ	>0.267	0.966	98.039	86.047	7.026	0.023	89.286	97.368
			(0.932–1.000)	(89.553–99.950)	(72.068–94.702)	(3.341–14.774)	(0.003–0.159)	(79.851–94.601)	(84.111–99.615)
	ADA + IFN-γ	>0.497	0.946	94.118	88.372	8.094	0.067	90.566	92.683
			(0.896–0.996)	(83.758–98.770)	(74.917–96.115)	(3.541–18.504)	(0.022–0.201)	(80.766–95.642)	(80.781–97.447)

TPE, tuberculous pleural effusion; non-TPE, non-tuberculous pleural effusion. HGF, hepatocyte growth factor; ADA, adenosine deaminase; IFN-γ, interferon gamma. M, male; F, female. AUC, area under the curve; PLR, positive likelihood ratio; NLR, negative likelihood ratio; PPV, positive predictive value; NPV, negative predictive value.

**Table 8:** Diagnostic performance of HGF + ADA, HGF + IFN-γ, and ADA + IFN-γ in PF in differentiating between patients with TPE and those with non-TPE according to female with no more than 65 years.

	Variable	Cut-off value	AUC (95% CI)	Sensitivity (%)	Specificity (%)	PLR	NLR	PPV (%)	NPV (%)
Age ≤ 65 y, F	HGF + ADA	>0.219	0.998	100	96.667	30	0	97.222	100
			(0.993–1.000)	(89.997–100.000)	(82.783–99.916)	(4.367–206.078)	-	(83.594–99.586)	-
	HGF + IFN-γ	>0.692	0.990	97.143	100	-	0.029	100	96.774
			(0.968–1.000)	(85.083–99.928)	(88.430–100.000)	-	(0.004–0.197)	-	(81.297–99.519)
	ADA + IFN-γ	>0.215	0.970	97.143	86.667	7.286	0.033	89.474	96.296
			(0.934–1.000)	(85.083–99.928)	(69.278–96.245)	(2.921–18.174)	(0.005–0.229)	(77.311–95.496)	(78.938–99.449)

TPE, tuberculous pleural effusion; non-TPE, non-tuberculous pleural effusion. HGF, hepatocyte growth factor; ADA, adenosine deaminase; IFN-γ, interferon gamma. M, male; F, female. AUC, area under the curve; PLR, positive likelihood ratio; NLR, negative likelihood ratio; PPV, positive predictive value; NPV, negative predictive value.

## Discussion

The World Health Organization (WHO)’s *Global tuberculosis report 2021* paints a rather grim picture of the trajectory of the global TB epidemic: a slow decline in incidence, but an increase in deaths due to TB. Not all of the objectives stated at the 2018 UN High Level Meeting on TB have been achieved (Chakaya et al., 2022). In addition, the pleura is a common locus of extrapulmonary TB (EPTB), with tuberculous pleurisy occurring in about 50% of EPTB patients (Kang et al., 2020; Shaw and Koegelenberg, 2021). Early animal investigations imply that delayed hypersensitivity brought on by *Mtb* infiltrating into the chest cavity, rather than a direct infection-induced local inflammatory response, is what causes tuberculous pleurisy (Chakrabarti and Davies, 2006; Shaw et al., 2019; Shaw and Koegelenberg, 2021). In addition, the bacterial load of *Mtb* in PE is often low, making the diagnosis of TPE more difficult and often requiring invasive surgery to obtain sufficient pleural tissue for histological and microbiological examination to confirm the diagnosis (Wang et al., 2015; Carlucci et al., 2019). Considering that invasive chest examination causes some harm to patients, it is valuable to explore soluble biomarkers for the diagnosis of TPE (Wang et al., 2015; Wang W. et al., 2018; Zhang et al., 2020).

In this study, we found that HGF expression was significantly increased in TPE. Further subgrouping by age and gender showed that it had better diagnostic efficacy in distinguishing TPE from non-TPE in female patients aged ≤65 years, with an AUC of 0.988, which was better than the diagnostic efficacy of either ADA or IFN-γ.

We also found that the expression of HGF in the pleural fluid from the TPE group was significantly higher than that of the non-TPE group, suggesting that HGF could play an important regulatory role. Previous studies have reported that age and gender might be independent factors strongly influencing the occurrence and development of diseases. Therefore, we divided TPE and non-TPE patients into age subgroups of ≤65 years and > 65 years. We found that HGF expression in the age ≤ 65 years TPE subgroup was higher than that in the corresponding non-TPE subgroup, and in ROC analysis, the AUC of HGF was higher in the TPE group than in the age > 65 years non-TPE subgroup. Meanwhile, in female TPE patients aged ≤65 years, the AUCs of TPE and non-TPE diagnosed by ADA or IFN-γ were 0.964 and 0.827, respectively. In TPE patients aged ≤65 years, male TPE patients aged ≤65 years or female TPE patients aged ≤65 years, the AUCs of ADA or IFN-γ did not differ significantly, fluctuating between 0.927–0.964 and 0.827–0.869, respectively. In particular, the AUC of female TPE patients aged ≤65 years reached 0.988, which was also higher than those of the corresponding ADA and IFN-γ subgroups.

According to our data, HGF in the PE of younger female patients had a PLR of 14.571, indicating that the probability of positive HGF was 13.571-fold higher in TPE patients than in non-TPE patients, which was high enough for diagnosis. Moreover, an NLR of 0.031 suggested that if the HGF result was negative, the probability of the patient being confirmed to have TPE was 3.1%, which was an acceptable value for ruling out TPE. The high PPV (94.444) and NPV (96.552) of HGF found in this study further indicated that both the false-negative and false-positive rates were low. However, the PLR, NLR, PPV, and NPV of ADA and IFN-γ in PE were less effective than those of HGF, suggesting that HGF had better diagnostic efficacy than ADA or IFN-γ in female TPE patients aged ≤65 years.

In our research, we found that combining multiple factors for diagnosis could improve diagnostic efficacy and help improve differential diagnosis between TPE and non-TPE. Our previous studies showed that the AUC of ADA plus interleukin-32 (IL-32) for the diagnosis of TPE was 0.994, and its SEN, SPE, PLR, NLR, PPV, and NPV were 93.0, 98.4%, 56.7, 0.1, 97.6, and 95.2%, respectively (Du et al., 2022). The SEN, SPE, PLR, NLR, PPV, and NPV of ADA plus IL-27 in the diagnosis of TPE were 91.3, 100%, 140.5, 0.09, 100, and 87.1%, respectively (Wu et al., 2013). In all subgroups, the diagnostic efficacy of HGF plus ADA or HGF plus IFN-γ was significantly higher; in the females aged ≤65 years group, the AUC of HGF plus ADA was the best, reaching 0.998. The AUC of HGF plus ADA in the diagnosis of TPE, meanwhile, was comparable to that of IL-27 plus ADA or IL-32 plus ADA.

Research has shown that age and sex play significant roles in immune system regulation (Markle and Fish, 2014; Giefing-Kröll et al., 2015). In addition, immune cells such as liver-derived macrophages can massively express and secrete HGF, thereby regulating hepatocytic regeneration and repair (Dong et al., 2019; Huang et al., 2022). These findings suggest that age or sex might help regulate the expression and secretion of HGF. In this study, HGF showed differential-diagnostic efficacy between TPE and non-TPE in different age and gender subgroups. In female patients aged ≤65 years, HGF or HGF plus ADA had markedly greater diagnostic efficacy, indicating that in TPE, HGF expression was also affected by gender and age. The specific mechanism of age and/or gender in regulating the expression of HGF needs further study.

This study has some limitations. First, the numbers of older patients with TPE and younger patients with non-TPE we included were too small, which was likely to have led to selection bias. Second, non-TPE subjects were mainly patients with malignant tumors, some benign tumors, and pneumonia; our sample lacked patients with PE caused by autoimmune diseases. In future studies, we will consider increasing the sample size, especially to include older TPE and younger non-TPE patients, so that our results will be more consistent with real-world statistics.

In conclusion, our study showed that the expression of HGF in PE was significantly higher in TPE patients than in non-TPE patients and that it had favorable diagnostic efficacy in female TPE patients aged ≤65 years, even better than that of ADA or IFN-γ.

## Data availability statement

The raw data supporting the conclusions of this article will be made available by the authors, without undue reservation.

## Ethics statement

The studies involving human participants were reviewed and approved by the Ethics Committee of Beijing Chao-Yang Hospital of CMU (2019-ke-37). The patients/participants provided their written informed consent to participate in this study. Written informed consent was obtained from the individual (s) for the publication of any potentially identifiable images or data included in this article.

## Author contributions

KZ: conceptualization. S-CZ, Z-YH, H-ZS, and M-MS: data curation, formal analysis, methodology, and validation. H-ZS: funding acquisition, investigation, and supervision. H-ZS and M-MS: project administration. S-CZ and M-MS: software and visualization. S-CZ: writing – original draft. S-CZ and KZ: writing – review and editing. All authors contributed to the article and approved the submitted version.

## Funding

This work was supported in part by grants from the National Natural Science Foundation of China (No. 81970088) and the Beijing Scholars Program (No. 048).

## Conflict of interest

The authors declare that the research was conducted in the absence of any commercial or financial relationships that could be construed as a potential conflict of interest.

## Publisher’s note

All claims expressed in this article are solely those of the authors and do not necessarily represent those of their affiliated organizations, or those of the publisher, the editors and the reviewers. Any product that may be evaluated in this article, or claim that may be made by its manufacturer, is not guaranteed or endorsed by the publisher.
