# Supplementary material for: Hepatocyte growth factor combined with adenosine deaminase as biomarker for diagnosis of tuberculous pleural effusion
Source: Front Microbiol. 2023 Jul 6;14:1181912. doi: 10.3389/fmicb.2023.1181912 (PMC10359098; doi:10.3389/fmicb.2023.1181912)
Supplement: Supplementary file 1 [file Data_Sheet_1.docx]

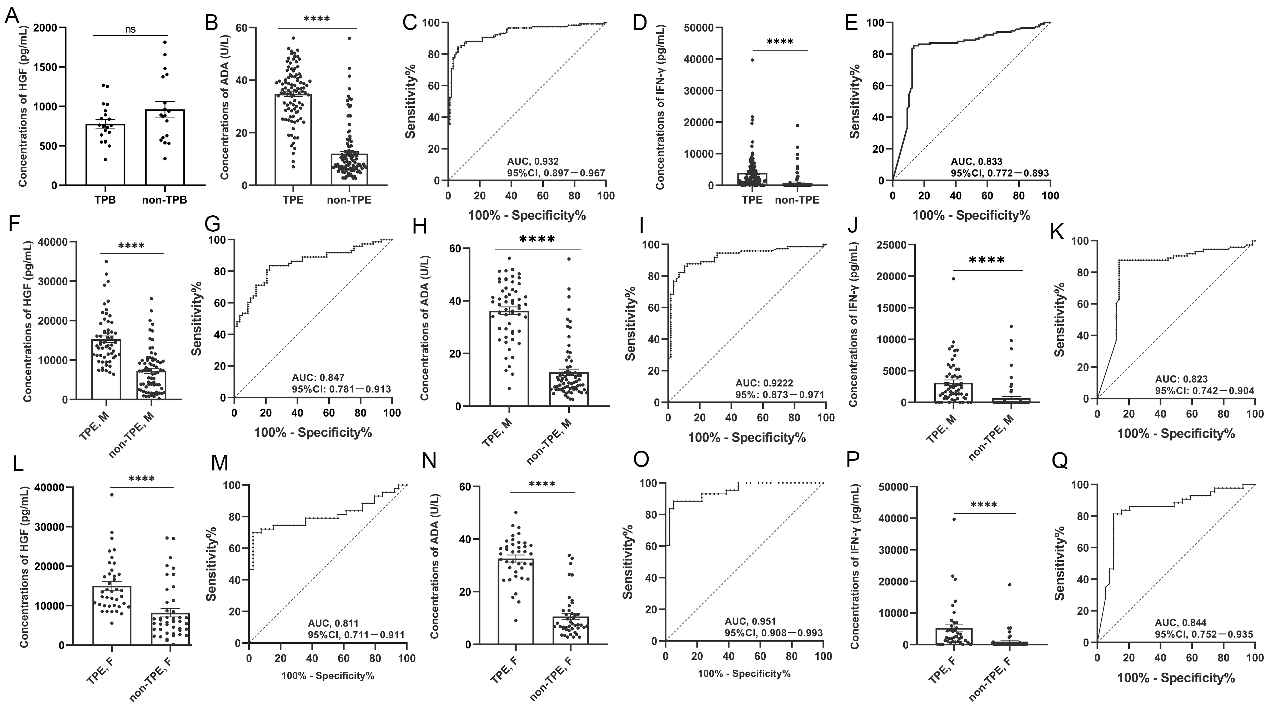


**Figure S1.** Diagnostic accuracy of HGF, ADA, and IFN-γ in PBs or PE for TPE overall or by gender. Expression of HGF in PBs from TPE and non-TPE patients **(A)**; expression of HGF, ADA, and IFN-γ in PE from TPE and non-TPE patients overall or by gender, respectively **(B**, **D**, **F**, **H**, **J**, **L**, **N**, and **P)**; ROC curves show the diagnostic value of HGF, ADA, and IFN-γ in TPE and non-TPE patients overall or by gender, respectively (**C**, **E**, **G**, **I**, **K**, **M**, **O**, and **Q**). **P* < 0.05, ***P* < 0.01, ****P* < 0.001, *****P* < 0.0001. TPE, tuberculous pleural effusion; non-TPE, non-tuberculous pleural effusion. HGF, hepatocyte growth factor; ADA, adenosine deaminase; IFN-γ, interferon gamma. M, male; F, female.


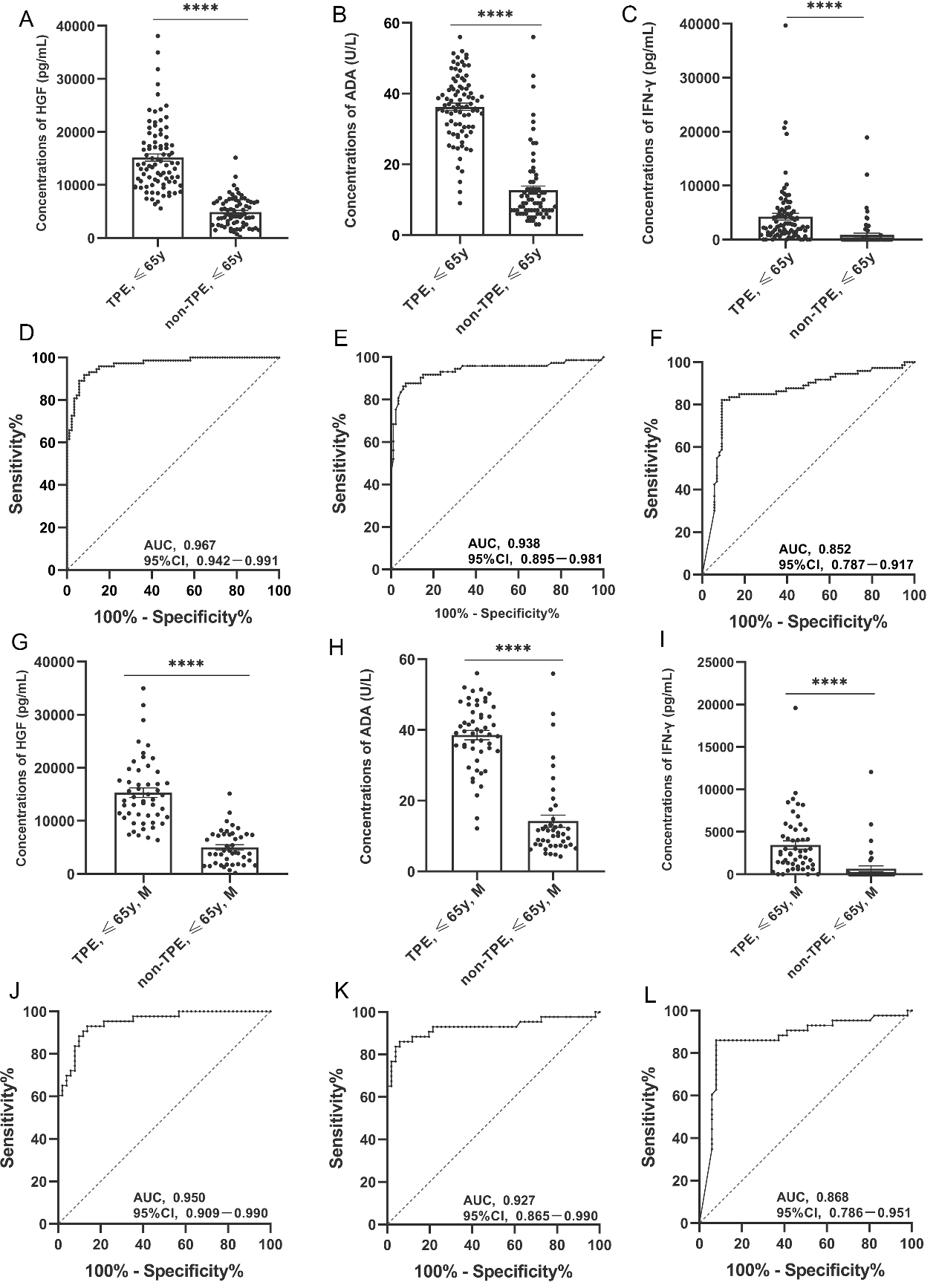


**Figure S2.** Expression and diagnostic accuracy of HGF, ADA, and IFN-γ in PE for TPE overall or by gender. Expression of HGF in PE from TPE and non-TPE patients aged ≤65 years **(A–C)**; AUC value of HGF in PE from TPE and non-TPE patients aged ≤65 years **(D–F)**; expression of HGF in PE from male TPE and non-TPE patients aged ≤65 years **(G–I)**; AUC value of HGF in PE from male TPE and non-TPE patients aged ≤65 years **(J–L)**. **P* < 0.05, ***P* < 0.01, ****P* < 0.001, *****P* < 0.0001. TPE, tuberculous pleural effusion; non-TPE, non-tuberculous pleural effusion. HGF, hepatocyte growth factor; ADA, adenosine deaminase; IFN-γ, interferon gamma. M, male; F, female.
